# Federated Machine Learning for Skin Lesion Diagnosis: An Asynchronous and Weighted Approach

**DOI:** 10.3390/diagnostics13111964

**Published:** 2023-06-05

**Authors:** Muhammad Mateen Yaqoob, Musleh Alsulami, Muhammad Amir Khan, Deafallah Alsadie, Abdul Khader Jilani Saudagar, Mohammed AlKhathami

**Affiliations:** 1 Department of Computer Science, Abbottabad Campus, COMSATS University Islamabad, Abbottabad 22060, Pakistan; mateenyaqoob@gmail.com; 2 Information Systems Department, Umm Al-Qura University, Makkah 21961, Saudi Arabia; dbsadie@uqu.edu.sa; 3 Information Systems Department, College of Computer and Information Sciences, Imam Mohammad Ibn Saud Islamic University (IMSIU), Riyadh 11432, Saudi Arabia; aksaudagar@imamu.edu.sa (A.K.J.S.); maalkhathami@imamu.edu.sa (M.A.)

**Keywords:** skin cancer prediction, privacy aware machine learning, federated learning for skin lesion, distributed machine learning, privacy in healthcare, privacy-aware image processing

## Abstract

The accurate and timely diagnosis of skin cancer is crucial as it can be a life-threatening disease. However, the implementation of traditional machine learning algorithms in healthcare settings is faced with significant challenges due to data privacy concerns. To tackle this issue, we propose a privacy-aware machine learning approach for skin cancer detection that utilizes asynchronous federated learning and convolutional neural networks (CNNs). Our method optimizes communication rounds by dividing the CNN layers into shallow and deep layers, with the shallow layers being updated more frequently. In order to enhance the accuracy and convergence of the central model, we introduce a temporally weighted aggregation approach that takes advantage of previously trained local models. Our approach is evaluated on a skin cancer dataset, and the results show that it outperforms existing methods in terms of accuracy and communication cost. Specifically, our approach achieves a higher accuracy rate while requiring fewer communication rounds. The results suggest that our proposed method can be a promising solution for improving skin cancer diagnosis while also addressing data privacy concerns in healthcare settings.

## 1. Introduction

Artificial intelligence (AI) has become increasingly popular in recent years as a powerful tool for solving various modern problems. One of the branches of AI is machine learning (ML), which enables computers to learn from data without explicit programming. Deep learning (DL) is a type of ML that uses artificial neural networks (ANNs) to learn models and patterns instead of being specifically programmed for a task. Many medical fields have adopted machine learning and deep learning methods for identifying different disorders.

However, collecting sensitive health information, such as patients’ confidential data, can be challenging due to privacy concerns. Health institutions may need to transfer data to a server for training an ML or DL model, which creates a potential risk of compromising patients’ privacy and security. To address this concern, several international regulations such as the California Consumer Privacy Act (CCPA) [1], General Data Protection Regulation (GDPR) [2], and Cybersecurity Law of China [3] have been proposed to ensure the privacy of clients’ data.

**Figure 1:**
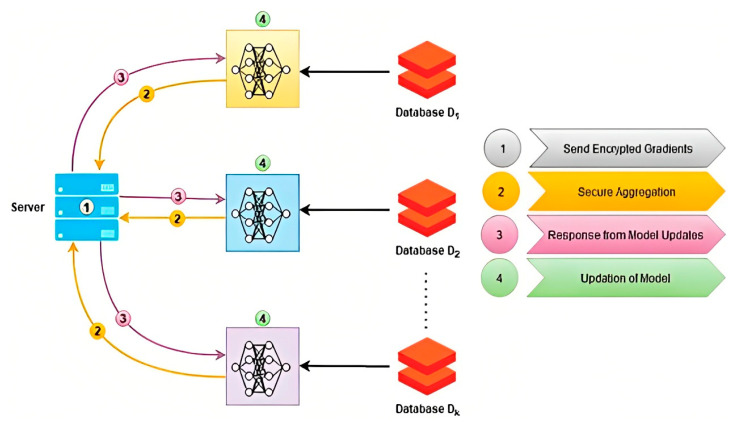
Federated learning and its working mechanism.

To overcome this challenge, federated learning (FL) is an excellent technique. FL allows a shared global model to be trained on local clients’ parameters without disclosing sensitive data to the server [4]. The clients train their local models on their private data, and the trained models are then aggregated on the server to create a global model. Figure 1 depicts the working mechanism of FL in a healthcare system. This global model is then sent back to the clients to train their models on. FL is critical for maintaining patient privacy and security while achieving clinical-grade accuracy, as shown by various workflows in the health field [5].

Healthcare data are very sensitive, and their use is tightly controlled due to the significant time, effort, and cost associated with collecting, curating, and maintaining a high-quality dataset. Therefore, data sharing is uncommon in the industry, resulting in cooperative algorithm training without data exchange. In FL workflows, aggregation can take place on a server or through peer-to-peer sharing. Federated learning requires a lot of communication resources, but the FedOpt approach offers a solution by using the Sparse Compression Algorithm (SCA) to reduce communication overhead [6]. FedOpt also uses lightweight homomorphic encryption to aggregate gradients securely and effectively. To maintain privacy and uniqueness, a differential privacy strategy based on the Laplace mechanism is utilized.

Synchronous federated learning involves clients sending their models or gradients to the server, and aggregation begins after all clients have sent their models. Asynchronous federated learning reduces communication costs by dividing models into shallow and deep layers that use CNNs, DNNs, or LSTMs. In ref. [7], asynchronous federated learning with a temporal-weighted aggregation approach is proposed to further reduce communication costs. Their contributions include an asynchronous technique that updates parameters in the shallow and deep layers of CNNs at various rates to reduce the number of parameters that need to be sent to the server. Additionally, a temporally weighted aggregation approach is suggested to aggregate trained models at the client level more effectively.

Skin cancer is a disease characterized by uncontrolled growth of certain body cells that spread to other parts of the body. Skin cancer can be caused by the abnormal growth of skin cells and is usually found in areas of the skin that are exposed to the sun, such as the face, arms, neck, or hands. There are three main types of skin cancer, including basal cell carcinoma, squamous cell carcinoma, and melanoma.

**Figure 2:**
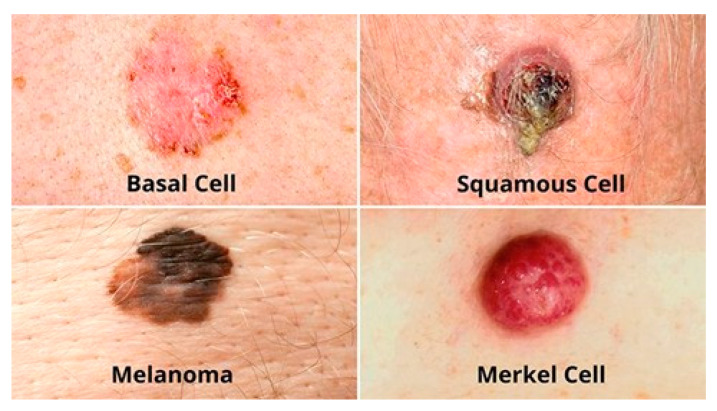
Types of skin lesions.

Melanoma is the most dangerous and serious type of skin cancer, which occurs due to severe damage and mutation of skin cells’ DNA. The damaged skin cells replicate quickly and form malignant tumors due to exposure to UV light or the use of artificial tanning tools. If melanoma is detected at an early stage, it is treatable. Basal cell carcinoma is the most common form of skin cancer and affects basal cells, which are in the deeper layers of the epidermis, and it is primarily caused by significant sun exposure to certain body areas. Squamous cell carcinoma (SCC) can develop on any part of the skin, including the mouth and genital mucous membranes, but it is more commonly found in areas such as the ears, lower lip, face, bald area of the skull, neck, hands, arms, and legs. The skin in those areas often shows obvious signs of solar damage, including wrinkles, color changes, and loss of flexibility. Figure 2 shows these types of skin lesions.

Early detection is critical in treating skin cancer, and melanoma is the most dangerous type, responsible for up to 75% of deaths. Researchers in [8] developed a deep learning method for accurately detecting skin cancer using 11 candidate single architectures of convolutional neural networks (CNNs) on the HAM10000 dataset, which contains 7 categories of cutaneous lesions. They addressed issues with imbalances and similarity between skin lesion photos by using data augmentation, transfer learning, and fine-tuning. The model achieved a 92% accuracy rate. In ref. [9], a fully automated technique was proposed for segmenting cutaneous melanoma at the earliest stage using a combination of deep learning techniques, including faster region-based convolutional neural networks (RCNN) and fuzzy k-means (FKM) clustering. The proposed method was tested using various clinical photos to determine whether it could help dermatologists diagnose this potentially fatal condition early. The presented technique performs preprocessing on the dataset photos to eliminate noise and lighting issues and improve visual information before using faster-RCNN to produce a feature vector of fixed length. FKM is then used to divide the melanoma-affected skin area into segments with varying sizes and borders.

The issues of privacy and communication efficiency motivate us to propose an asynchronous FL technique using CNN for skin cancer prediction. This proposed technique allows local training on many distributed devices without requiring them to be synchronized, which is particularly useful for mobile devices with limited connectivity. This approach can leverage a large amount of data from different sources to train a more accurate model while preserving data privacy. Asynchronous federated learning is also robust to network failures and can continue to learn from available devices, making it a promising approach for skin cancer prediction. Our paper proposes a decentralized privacy-aware method for skin cancer prediction in a healthcare environment. The key contributions of our approach are as follows:

- We introduce a novel approach that improves communication efficiency and prediction accuracy for skin cancer prediction, addressing data privacy concerns in healthcare settings.
- We leverage convolutional neural networks (CNNs) for client ends and an asynchronous version of federated learning (FL) for model aggregation at the global center server. This approach enables us to optimize communication rounds by dividing CNN layers into shallow and deep layers, with shallow layers updated more frequently.
- We evaluate our approach on a skin cancer dataset and show that it outperforms existing methods and baseline FL methods in terms of convergence time, prediction accuracy, and communication efficiency for skin cancer prediction. Specifically, our approach achieves a higher accuracy rate while requiring fewer communication rounds.

Our results demonstrate that our proposed method can provide a promising solution for improving skin cancer diagnosis while addressing data privacy concerns in healthcare settings. This contribution can potentially impact the development of similar privacy-aware machine learning methods for healthcare applications. Section 2 discusses related work in skin cancer detection using DL, ML, and AI methods. Section 3 gives the proposed methodology. Section 4 is about the results of the proposed methodology, and finally, Section 5 concludes the article.

## 2. Related Work

From environmental causes to hereditary vulnerability, there are many elements that affect how skin disorders are influenced. Many socioeconomic issues, including wealth, inequality, poverty and education, and access to medical care, are responsible. The Global Burden of Disease (GBD) study [10] found that skin conditions ranked fourth among the most prevalent diseases in terms of the burden they place on society. Both in high- and low-income nations, skin conditions are a major contributor to psychological and social difficulties [11]. 

Anxiety, despair, rage, social isolation, and low self-esteem are among symptoms of skin disease [12,13]. If skin illness is discovered before it has been present for a long time, it is expected that it will be treatable. Dermatologists struggle to identify skin illnesses; nevertheless, many of them share the same color and anatomical features [14]. However, ML has made a startling change possible. ML has made medical imaging dramatically different, particularly in terms of illness identification. ML models have demonstrated activity in medical research at the level of humans thanks to advancements in computer processing power and the availability of an enormous amount of data [15]. For instance, CNNs have sped up the development of medical image processing (such as CT and MRI scans) [16]. Clinical photos are unsuitable for study because of varying resolutions, complex situations, and privacy issues, especially with images of delicate body parts. Additionally, the dataset image for skin diseases is not well labelled with information. Furthermore, there are extremely few accessible datasets that contain labelled data. For all disorders, research on skin pictures is problematic. 

For the diagnosis and classification of skin diseases, numerous research publications have been published. Many of these researchers have used deep learning algorithms to categorize skin diseases [17]. For instance, ref. [18]’s use of the V3 inception design shows good accuracy in classifying skin cancers. For 9 kinds of malignancies, 2 dermatological tests had respective accuracies of 55.0% and 53.3%. A 55.4% average accuracy was displayed by the model. They investigated a nonlinear support vector machine (SVM) method for melanoma diagnosis, using 70% of the data for training and 30% for testing. The studies’ average accuracy rate was 76%. Zhang et al., in 2018 [19], also suggested the V3 inception design on dermoscopy images utilizing the same network for classifying four common skin diseases, including melanocytic nevus, psoriasis, SK, and BCC. The results were 87.25% accurate. Rarely are the studies mentioned above more than 90% accurate. Photos of diabetic retinopathy from the Messidor dataset were categorized using a modified AlexNet architecture in [20]. Based on four categories, they have been divided as follows: healthy retina, DR stage 1, DR stage 2, and DR stage 3. Both the DR stage 1 and the DR stage 3 displayed a maximum accuracy of 96.6% for the AlexNet model. To identify skin conditions, viral infections, and bacterial infections, Tushabe et al. investigated various machine learning techniques, including the twin-layer perceptron neural network (NN), the k-nearest neighbor classifier (KNN), and the support vector classifier with two norms (SVC) [21]. After the KNN classifier, which showed maximum accuracy, the SVM classifier showed a higher accuracy of 92%. Despite receiving the maximum level of accuracy, some crucial measures (f1 score, recall, and precision) are not described. It is also unknown how many datasets were used. 

The authors of [22] suggested classification research to diagnose melanoma based on dermoscopy images using a multilayer preceptor (MLP) classifier. Higher training and testing accuracy were shown by the MLP. Gurovich et al. [23] introduced a CNN method known as DeepGestalt, and the model was trained using 17,000 face photographs of people with genetic diseases. More than 200 images can be consistently and precisely identified by their program. To the best of our knowledge, there has not been a model created for classifying skin diseases that takes the privacy of the photographs into account. 

The use of FL in the field of medicine is being attempted by several researchers. Researchers in [24] demonstrated a federated learning system that can create a global model from local data spread over many sites that are stored locally. They demonstrated the practicality and efficacy of the FL architecture using actual electronic health data from more than 1 million patients, while keeping the relevance of the global model. In ref. [25], four types of skin diseases were included in a custom image dataset, a CNN model was proposed and compared with multiple CNN algorithms, and an experiment was run to test how well data privacy could be maintained utilizing an FL strategy. The dataset was expanded, and the model was made broader using an image augmentation method. The concept of classifying human skin using CNN-based skin disease categorization combined with FL is astounding. Utilizing four different publicly available datasets, the performance and generalization capabilities of the proposed system are evaluated when several heartbeat classifications are considered. The experimental findings show that the suggested asynchronous federated learning (Async-FL) approach can achieve favorable classification performance while simultaneously ensuring privacy, flexibility to varied subjects, and reducing network bandwidth usage [26]. 

Classification of skin cancer using an improved NN-based technique is proposed in [27]. They utilized transfer learning and LSTM for effective classification of skin lesions. Skin cancer detection using an enhanced version of FL is proposed in [28,29]. To evaluate the effectiveness of FL for healthcare service providers, the authors in [30,31] propose the upgraded versions of FL for effective disease diagnosis, while ensuring data privacy and achieving better prediction accuracy. A solution to optimization problems using a distributed algorithm is proposed in [32]. They proposed a DANE algorithm based on the Newton-type distributed optimization algorithm, which aims to minimize the communication rounds among the nodes. 

## 3. Materials and Methods

In this section, an enhanced FL approach with CNN is proposed to address the issues of privacy and effective prediction of skin lesion in a healthcare system. Skin cancer is a condition that can be fatal. We proposed a model employing CNN and asynchronous version of FL for precise privacy-aware skin cancer prediction.

### Proposed Asynchronous FL-CNN for Skin Cancer

A CNN method is employed to identify skin cancer at the client level. The multiple layers that make up the CNN are individually used to extract features. Since shallow levels in a CNN learn the generic features that are relevant to numerous tasks and datasets, only a tiny percentage of the parameters in CNNs (those in the shallow layers) represent features general to different datasets. The number of communication rounds rises because of the constant updating of these layers for better outcomes. In this paper, an asynchronous method for federated learning is proposed, which allows multiple client nodes to train a model collaboratively. The main objective is to develop a model in such a manner that provides better accuracy across all clients. This proposed method reduces the communication rounds required by updating shallow layers more often than deep layers. In the traditional FL, all layers of the model are updated synchronously, hence resulting in a communication burden. Our proposed Async-FL-CNN method updates shallow layers concurrently with deep layers to reduce the amount of data that needs to be transferred. To classify images, the Async-FL-CNN method uses CNN architecture, which consists of convolutional layers, max-pooling layers, and fully connected layers. The cross-entropy loss function measures the difference between predicted and actual class labels. 

FL traditional algorithms face two primary issues, which are low accuracy and high communication overhead. High communication overhead results from the large number of communication rounds required to train the model. The Async-FL-CNN method addresses these issues in multiple ways. First, the layer-by-layer asynchronous updating method reduces the communication overhead by updating the shallow layer parameters more frequently than deep layer parameters. Second, the proposed method uses dropout, a regularization technique that prevents overfitting and improves generalization across clients. Finally, the proposed method uses a client selection algorithm that selects the most suitable clients based on their data and hardware characteristics, further improving the accuracy and efficiency of the model. 

**Figure 3:**
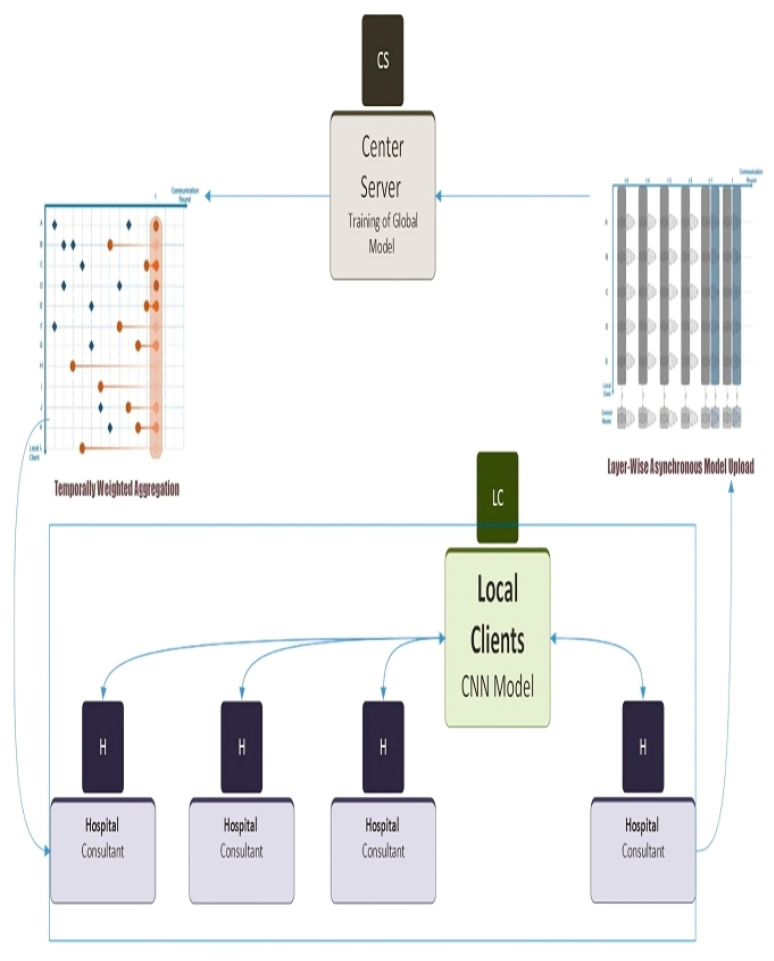
Overview of the proposed Async-FL-CNN method.

This is what we refer to as a layer-by-layer model update, and it is depicted in Figure 3.

**Figure 4:**
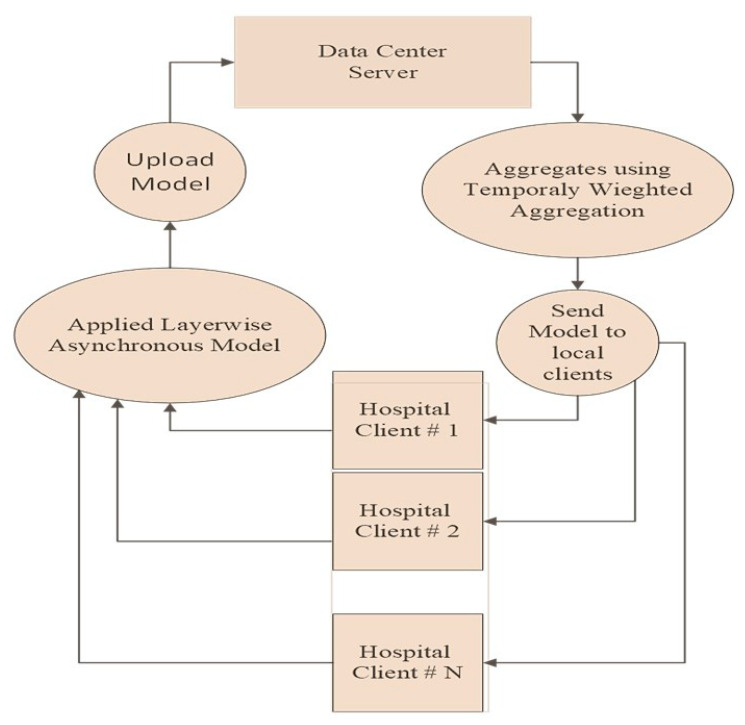
Working of proposed Async-FL-CNN method.

Algorithms 1 and 2 depict the stagewise working of the proposed method for both the client and center sever end. The proposed Async-FL-CNN works at the hospital client end and at the data center server end, and it is illustrated in Figure 4. The working of each stage is explained below.

Initialization: Async-FL starts by initializing a u_0_ global model, which is then shared among every *k* client.

Local Client Training: Each local client *k*, upon reception of initial u_0_ global model, trains the global model on its local data (skin cancer dermoscopy images) asynchronously, without waiting for other clients. Initially CNN function is applied for the detection of skin cancer. The subfunction gets k and u. B and E are the local mini-batch size and the number of local epochs, respectively. η is the learning rate. The updated models are sent to the central server.

Aggregation: In line 5 of Algorithm 1, the central server collects and aggregates the model updates from all the clients to create a new global model.

Model Update: The central server updates the global model with the aggregated updates and sends it back to all the clients.

Evaluation: Each client evaluates the global model on their local data and sends the results to the central server.

Termination: The training process continues until a maximum number of iterations. The final global model is then used for inference.
**Algorithm 1:** Center Server Asynchronous FL-CNN1: initialize with u_0_ as initial global model 2: Perform for every local client *k* ∈ {1, 2…, *K*} (i) Assign (*timestamp*_g_^*k*^, *timestamp*_s_^*k*^) ← 0 3: **for** each communication round = 1, 2…*t*
(i) if (*t* % *current round* = *set_ES_*) (ii) Assign *flag* = 1 (iii) else assign *flag* = 0 (iv) *m* ← maximum from (*C* and *K*) (v) *S_t_* ← (selection of random set of *m* clients) 4: **for** every *k* ∈ *S_t_*
**parallelly compute**
(i) if *flag* =1 (ii) u^*k*^ ← Client-Side-Function (*k*, u*_t_, f lag*) (iii) Update (*timestamp*_g_^*k*^ ← *t, timestamp*_s_^*k*^ ← *t)* (iv) else u_g_^*k*^ ← Client-Side-Function (*k*, u_*g*_, *t*, *flag*) 5: if *flag* = 1 then (i) u*_g_*_, *t* + 1_ ← ($\overset{K}{\underset{k=1}{\sum }}n_{k}/n$) * *f*_g_ (*t*, *k*) ∗ u^*k*^ //where *f*_g_ (*t*, *k*) = α − ^(t −^
*timestamp*_g_^*k*^^)^ (ii) else u*_s_*_, *t* + 1_ ← ($\overset{K}{\underset{k=1}{\sum }}n_{k}/n$) * *f*_s_ (*t*, *k*) ∗ u_s_ //where *f*_s_ (*t*, *k*) = α − ^(t −^
*timestamp*_s_^*k*^^)^

**Algorithm 2:** Client-Side Asynchronous FL-CNNClient-Side-Function (*k*, v, *f lag*) 1: for i = 1 to *k*
2: Convolution 3: Perform convolution 4: Average pooling with fully connected 5: ReLu function 6: B ← (split P*k* into batches of size *B*) 7: **for** each local epoch *i* from 1 to *E*
**do**
8: **for** batch *b* ∈ B **do**
9: u ← u_s_ − η ∗ (v; *b*) 10: **if flag** = 1 (i) return u to server (ii) **else** return u_s_ to server 

## 4. Experimental Results and Discussion

### 4.1. Description of Utilized Dataset

The ISIC-2019 dataset of dermoscopy skin lesion images was used. In this section, we discuss the dataset used for effective melanoma skin lesion detection and classification. We perform the training and testing of our proposed technique on International Skin Imaging Collaboration (ISIC) 2019 dataset. This ISIC-2019 dataset described in Table 1 (available at https://challenge2019.isic-archive.com/ accessed on 8 March 2023) contains skin lesion images from eight different classes of skin cancers, namely melanoma (MLA), basal cell carcinoma (BCC), squamous cell carcinoma (SCC), vascular lesion (VLN), melanocytic nevus (MCN), benign keratosis (BGK), actinic keratosis (ATK), and dermatofibroma (DFA). The ISIC-2019 dataset contains overall 25,331 dermoscopy images, 21,491 for training, 1930 for testing, and 1910 for validation. 

### 4.2. Experimental Implementation

The experimentation of the proposed method is conducted using the *PyTorch* programming library for machine learning tasks on Google Colaboratory with NVIDIA Tesla T4 16 GB graphic processing unit (GPU). Table 2 below describes the experimental settings and parameters used for the experiments. 

The federated learning setting requires data to be distributed among the clients for the local training, and our implementation is carried out for five clients. Details of the dermoscopy image data distributed among the clients are demonstrated in Table 3.

### 4.3. Results and Discussion

The performance of the proposed method in terms of accuracy, loss, precision, consumption of communication size, local epoch effects, and convergence rate is measured and compared with the existing baseline federated learning methods, such as the CNN version for FedAvg (Federated Averaging) and FedSGD (Federated Stochastic Gradient Descent), as well as with one existing technique, Fed-Ensemble-CNN. Table 4 depicts the performance of existing methods and our proposed technique in terms of F1 score, recall, sensitivity, specificity, precision, and loss. The proportion of correctly classified samples out of the total number of samples was used to measure accuracy.

(1) $$\mathrm{Accuracy}=\frac{\mathrm{TP}\;+\;\mathrm{TN}}{\left(\mathrm{TP}\;+\mathrm{TN}+\mathrm{FP}+\mathrm{FN}\right)}$$

whereas the proportion of true positives out of all positive samples was used to calculate sensitivity or recall.

(2) $$\mathrm{Recall}=\frac{\mathrm{TP}}{\left(\mathrm{TP}+\;\mathrm{FN}\right)}$$

The proportion of true positives out of all positive samples was used to calculate sensitivity or precision.

(3) $$\mathrm{Precision}=\frac{\mathrm{TP}}{\left(\mathrm{TP}+\;\mathrm{FP}\right)}$$

The fraction of true negatives in all negative samples was defined as specificity.

(4) $$\mathrm{Specificity}=\frac{\mathrm{TN}}{\left(\mathrm{TN}+\;\mathrm{FP}\right)}$$

The F1 score was determined as a mix of precision and recall.

(5) $$F1-\mathrm{Score}=2*\;(\frac{\mathrm{Precision}*\mathrm{Recall}}{\mathrm{Precision}+\;\mathrm{Recall}})$$

The training, validation, and testing accuracy of the proposed method is compared with existing methods and is shown in Table 5. Our proposed Async-FL-CNN achieves better performance as compared with the existing and baseline models. Because the proposed technique allows the local clients to asynchronously train their local datasets using the CNN. 

**Figure 5:**
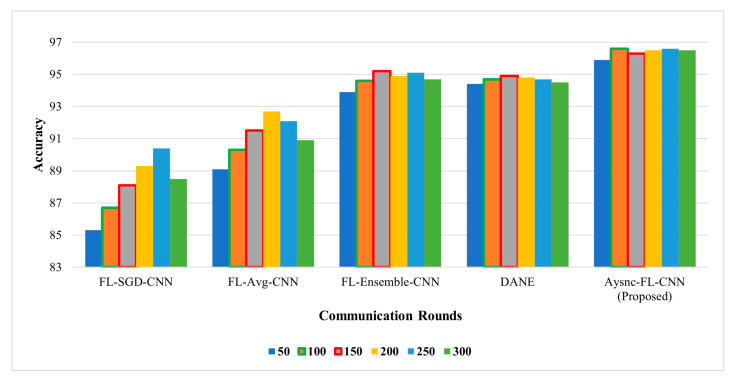
Effect of communication rounds on accuracy.

The convergence rate is defined in terms of rounds of communication utilized to achieve the accuracy of the model. Figure 5 illustrates the comparison of the convergence rate of the proposed method. Our proposed technique achieves higher accuracy with less communication rounds.

The ISIC-2019 dataset consists of eight classes of skin lesions. The classwise precision, specificity, sensitivity, and recall achieved by the proposed Async-FL-CNN is illustrated in Table 6. The proposed asynchronous federated learning approach with CNN achieved remarkable performance metrics for skin cancer prediction. The model demonstrated high accuracy, sensitivity, specificity, precision, and recall. The reported sensitivity of 94.1% and specificity of 96.3% indicate that the model accurately identified true positive and true negative cases. Additionally, the reported precision of 96.7% and recall of 92.6% suggest that the model correctly identified a high proportion of positive cases with a relatively low number of false positives. Overall, these performance metrics demonstrate that the proposed asynchronous federated learning approach with CNN was effective in achieving high accuracy and robustness for skin cancer prediction. 

**Figure 6:**
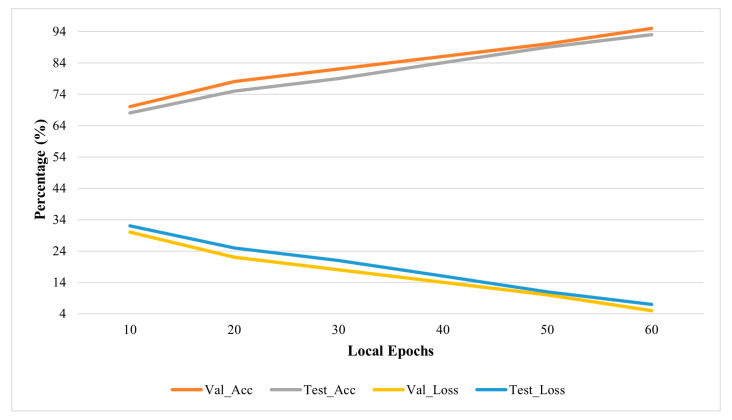
Comparison of validation and testing accuracy with loss for Async-FL-CNN.

Figure 6 depicts the comparison of validation and testing accuracy with the validation and testing loss of the proposed Async-FL-CNN method.

**Figure 7:**
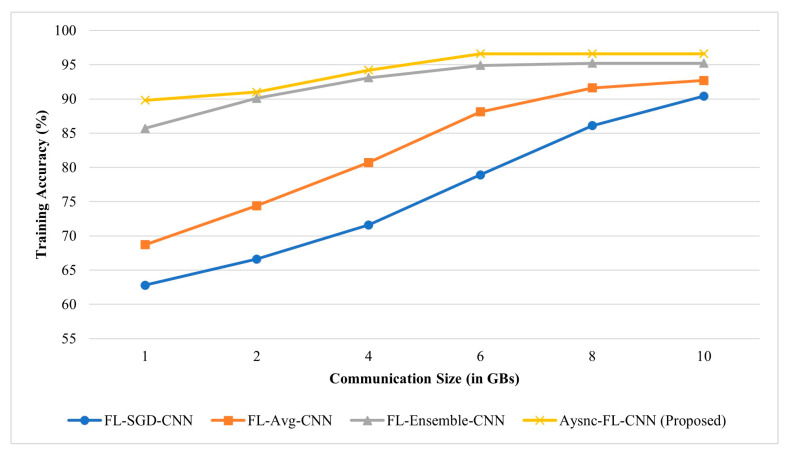
Communication efficiency comparison.

The communication efficiency in terms of consumed volume for communication by the methods is depicted in Figure 7.

## 5. Conclusions

This article proposes an asynchronous model update technique and a CNN method to detect skin cancer disease and to lower the communication costs and enhance the learning performance of federated learning. This article proposes an asynchronous learning strategy on the clients and a temporally distributed learning approach to present an improved federated learning technology: weighted aggregation of the server’s local models. The accuracy and convergence of the central model are improved by introducing an asynchronous weighted temporal approach on the server to utilize the previously trained local models on a dataset of skin lesions to test the proposed approach. This study makes the supposition that all local models use the same neural network design and share common hyperparameters, including the SGD learning rate. To further enhance learning performance and lower communication costs, we will build new federated learning algorithms in our next research that will enable clients to develop their own models.

## Figures and Tables

**Table 1:** Description of Skin Lesion Classes in ISIC-2019 dataset.

Class	Images for Training	Images for Testing	Images for Validation	Total Images
MLA	3812	360	350	4522
BCC	2820	250	253	3323
SCC	541	42	45	628
VLN	202	24	27	253
MCN	10,979	965	931	12,875
BGK	2215	203	206	2624
ATK	716	75	76	867
DFA	206	11	22	239

**Table 2:** Parameters Utilized for Experimental Implementations and Settings.

Parameter	Value
Simulation environment	Python
Utilized dataset	ISIC-2019
Client nodes	5
Communication rounds	1000
Epochs	60
Learning Rate	0.001
Size of mini batch	128
Optimizer	Adam
Activation function	ReLu
Size of communication	1 GB–10 GBs

**Table 3:** Client wise image distribution.

Clients	Number of Images in Each Class							
	MLA	BCC	SCC	VLN	MCN	BGK	ATK	DFA
1	904	664	126	51	2575	524	174	49
2	904	665	126	50	2575	525	174	48
3	904	665	125	50	2575	524	173	47
4	905	665	126	51	2575	526	173	48
5	905	664	125	51	2575	525	173	47

**Table 4:** Comparison of Performance with the Proposed Method.

Model	F1 Score	Sensitivity	Recall	Specificity	Loss	Precision
FL-SGD-CNN	88.7	90.1	90.3	91.4	5.1	92.8
FL-Avg-CNN	90.5	90.8	90.7	92.1	3.5	93.2
FL-Ensemble-CNN	94.2	93.6	91.1	95.4	2.5	95.3
DANE	94.1	93.7	91.5	95.7	3.4	95.8
Async-FL-CNN (Proposed)	94.8	94.1	92.6	96.3	1.6	96.7

**Table 5:** Comparison of Accuracies.

Model	Accuracy		
	Training	Validation	Testing
FL-SGD-CNN	90.4	85.3	80.2
FL-Avg-CNN	92.7	89.2	85.7
FL-Ensemble-CNN	95.2	92.7	90.2
DANE	94.9	91.5	89.8
Async-FL-CNN (Proposed)	96.6	95	93.4

**Table 6:** Classwise Achieved Precision and Recall.

Model	Classes of Skin Cancer	Precision	Recall	Sensitivity	Specificity
Aysnc-FL-CNN (Proposed)	MLA	99.1	93.4	98.1	98.7
	BCC	97.2	90.5	93.6	97.2
	SCC	96.6	93.1	90.2	96.1
	VLN	98.5	96.1	94.5	95.5
	MCN	99.8	98.5	94.2	96.3
	BGK	97.4	93.2	93.8	95.8
	ATK	97.3	91.5	95.5	96.4
	DFA	87.4	84.2	92.6	94.4

## Data Availability

We ran simulations to see how well the proposed approach performed. Any questions concerning the study in this publication are welcome and can be directed to the lead author (Muhammad Amir Khan) upon request.
